# Internal Migration Experience and Depressive Symptoms among Middle-Aged and Older Adults: Evidence from China

**DOI:** 10.3390/ijerph19010303

**Published:** 2021-12-28

**Authors:** Xiaodong Zheng, Yue Zhang, Yu Chen, Xiangming Fang

**Affiliations:** 1 School of Economics, Zhejiang Gongshang University, Hangzhou 310018, China; zhangyuezjsu@163.com; 2 Department of Agricultural and Applied Economics, The University of Georgia, Athens, GA 30602, USA; yc41981@gmail.com; 3 College of Economics and Management, China Agricultural University, Beijing 100083, China; xmfang@cau.edu.cn; 4 School of Public Health, Georgia State University, Atlanta, GA 30303, USA

**Keywords:** depressive symptoms, internal migration experience, middle-aged, elderly, China

## Abstract

Background: This study aimed to examine the association of internal migration experience with depressive symptoms among middle-aged and elderly Chinese, as well as explore possible mechanisms of the relationship. Methods: Participants were from the China Health and Retirement Longitudinal Study (CHARLS), a nationally representative sample of residents aged 45 years and older (*n* = 43,854). Survey data on depressive symptoms and internal migration experience were collected from biennial CHARLS surveys (CHARLS 2011/2013/2015) and a unique CHARLS life history survey in 2014, respectively. Multiple logistic regressions and the Karlson–Holm–Breen (KHB) method were employed in the statistical analyses. Results: The overall prevalence rate of depressive symptoms among middle-aged and older adults was 34.6%. Internal migration experience was associated with higher risks of depressive symptoms (OR = 1.07, 95% CI = 1.02–1.12, *p* < 0.01), especially among females (OR = 1.08, 95% CI = 1.01–1.14, *p* < 0.05), middle-aged adults (OR = 1.12, 95% CI = 1.06–1.19, *p* < 0.001), rural-to-urban migrants who had not obtained an urban *hukou* (OR = 1.13, 95% CI = 1.07–1.19, *p* < 0.001), and those who had low migration frequency and first migrated out at 35 years of age or older. Chronic disease (17.98%, *p* < 0.001), physical injury (7.04%, *p* < 0.001), medical expenditure (7.98%, *p* < 0.001), pension insurance (4.91%, *p* < 0.001), and parent–child interaction (4.45%, *p* < 0.01) were shown to mediate the association of internal migration experience with depressive symptoms. Conclusions: This study indicates that there is a significant association between internal migration experience and high risks of depression onset later in life. It is suggested to reduce institutional barriers for migrants and implement evidence-based interventions to improve migrants’ mental health.

## 1. Introduction

With the growing scope, complexity, and diversity of population movement, migration is becoming one of the determining global issues of the 21st century [1]. As the most populous country, China has witnessed large-scale internal population migrations since the last century, including migrations due to war, famine, send-down movement before the 1980s, and the massive rural-to-urban and urban-to-urban migrations during the past few decades. According to the seventh national census of China in 2020, there were 376 million internal migrants whose residences were not the places of household registration (*hukou*), with 249 million rural-to-urban migrants and 127 million urban-to-urban migrants, respectively [2]. Although migrating to urban areas is often beneficial to promote individual socioeconomic status in comparison with nonmigrants in the place of origin, city life is also widely considered as stressful because “cities are polluted, unhealthy, tiring, overwhelming, confusing, alienating” [3]. They are also “the places of low-wage work, insecurity, poor living conditions and dejected isolation” for the disadvantaged groups, such as the nonnative permanent residents [4,5]. This is also true for China’s internal migrants who have encountered challenges of institutional barriers (e.g., *hukou* system) and acculturation problems [6,7,8]. Some existing evidence has documented that internal migration contributes to the mental disorders among China’s rural-to-urban migrants [9,10]. However, few studies have investigated the association and its mechanisms between internal migration experience and psychological wellbeing from the life-course perspective. Research on these issues is important to the understanding of the long-term mental health consequences of the massive internal migration in China and other similar developing countries, as well.

The accumulation of the risk model and the pathway model are two crucial components of the life-course theory, which is often perceived as a conceptual framework to explain associations of early-life experiences with later-life outcomes [11,12]. The accumulation of risk model posits that later-life outcomes are formed in an accumulative pattern and the risks for human wellbeing tend to occur in clusters. The clustered deleterious exposures at different life-course stages can inflict accumulative risks and directly predict poor outcomes in later life [13,14]. The pathway model indicates that early-life conditions can indirectly affect later-life wellbeing through chains of risks or a series of linked adversities (mediating factors) that interact and aggregate with each other across the life span [15,16]. The above two theoretical models suggest that both direct and indirect effects should be of concern when investigating the association between internal migration experience and migrants’ mental health.

The household registration (*hukou*) system has long been the main institutional constraint for internal migrants in China. On the one hand, the *hukou* system has been closely associated with local social programs and resources, such as health care access and retirement pension [17,18]. As a result, China’s migrant workers, especially the low-skilled rural-to-urban migrants, are disproportionately employed in 3D jobs—dangerous, dirty, and demanding [19]. The physically demanding job and poor work environment may induce migrants’ chronic diseases and physical injuries, which subsequently increase the risk of mental illness. Furthermore, due to the limited access to health care and the poor flexibility of medical insurance transferability, migrants may have to bear more burden of medical expenses without reimbursement, which can also contribute to their life pressure and emotional problems [20,21,22]. In addition, with the lack of social pension insurance, migrants might also develop negative emotions, such as anxiety and depression, when considering their living quality after they become old [23,24]. This is particularly the case for internal migrants who are employed in 3D jobs for a long time with relatively low levels of working skills and heavy family financial burden [25]. On the other hand, the *hukou* system can also result in a split-household arrangement in which migrants work and live in cities while their family members (e.g., children) are left behind in home communities [26,27,28]. The separation of family members often leads to the absence of intra-household emotional support such as parent–child interactions, which in turn affects the psychological wellbeing of migrants. This negative effect is likely to be more prominent for those who have suffered long-term family separation [29].

The purpose of this study was to examine the association between internal migration experience and the presence of depressive symptoms among middle-aged and older adults. Differences in the mental health effects of migration experience by gender, age, and type of *hukou* were also investigated. Given that females are more susceptible to the risk factors for mental health than males [30,31], we assumed that the association of internal migration experience with depression was more salient for females. In addition, provided that middle-aged adults are more likely to be employed in high-intensity jobs and may suffer from more physical health loss than the older cohorts [32], the adverse mental health consequences might be more significant for middle-aged people. Compared to urban-to-urban migrants, rural-to-urban migrant workers are often less educated due to lower levels of household income and educational resources [33]. As a result, rural-to-urban migrant workers are more likely to be employed in 3D jobs and they are more prone to experience physical injury, chronic disease, long-term family separation, and consequent mental disorders, especially among the rural migrants who have not changed their rural *hukou* to an urban one. In addition, we explored potential mechanisms underlying the relationship between internal migration experience and depressive symptoms, including physical health (e.g., chronic diseases, physical injuries), social security (e.g., medical expenditure, pension insurance), and emotional support (parent–child interaction).

Accordingly, we proposed three hypotheses as follows:

**Hypothesis** **1** **(H1).** *The internal migration experience is adversely associated with depressive symptoms among middle-aged and older adults.*

**Hypothesis** **2** **(H2).** *The association between internal migration experience and depressive symptoms is more significant for females, middle-aged adults, and rural-to-urban migrants who have not obtained an urban hukou.*

**Hypothesis** **3** **(H3).** *Chronic disease, physical injury, medical expenditure, pension insurance, and parent–child interaction mediate the association of internal migration experience with depressive symptoms.*

## 2. Methods

### 2.1. Participants

This study drew data from the China Health and Retirement Longitudinal Study (CHARLS). CHARLS is a nationally representative longitudinal survey for Chinese residents aged 45 and older to support research on middle-aged and elderly Chinese. The survey is designed after the Health and Retirement Study (HRS) in the U.S., and it adopts a multistage, stratified, proportional-to-size (PPS) sampling process. The baseline wave of CHARLS was launched in 2011 to cover 28 of the 34 administrative divisions. It involved over 10,000 households and 17,500 individuals in 150 counties/districts and 450 villages/resident committees [34]. The 2013 and 2015 waves of CHARLS followed up with the baseline respondents and added new participants to compensate for the loss to follow-up. In addition, CHARLS conducted a unique life history survey in 2014, from which we could draw information on participants’ migration experience and other early-life conditions. According to the China Health and Retirement Report by the CHARLS team [35], the follow-up rates in 2013, 2014, and 2015 were 88%, 86%, and 87%, respectively. The ethnics application for the data collection was approved by the Biomedical Ethics Review Committee of Peking University (IRB00001052-11015). Ethnics application for the use of CHARLS data was approved by the University of Newcastle Human Research Ethics Committee (H-2015-0290).

**Figure 1:**
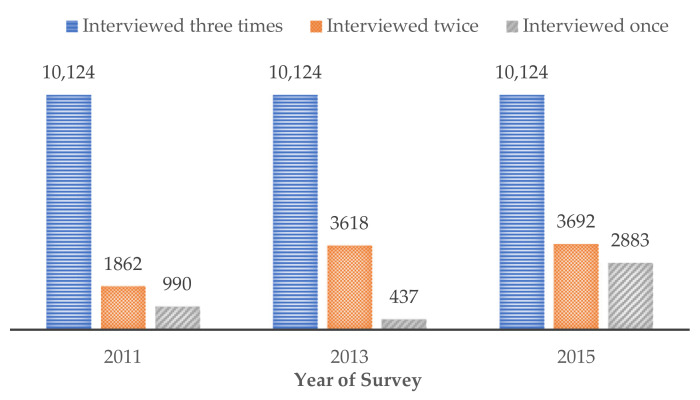
Distribution of the number of observations among CHARLS surveys.

To better control for the effect of time on depression, our study used data from the baseline CHARLS survey in 2011 and follow-up surveys in 2013 and 2015, as well as the 2014 life history survey, to investigate the association between internal migration experience and depressive symptoms among middle-aged (45–64 years) and older adults (65 years and older). We imposed two sample restrictions. First, we excluded observations (about 12%) that could not be matched with participants in the 2014 life history survey due to loss to follow-up. Second, we excluded missing observations (about 3%) on depressive symptoms, internal migration experience, and other study covariates. Finally, our sample included a total of 43,854 observations, with 10,124 participants interviewed three times (10,124 × 3 = 30,372 observations), 4586 participants surveyed twice (4586 × 2 = 9172 observations), and 4310 participants surveyed once (4310 × 1 = 4310 observations). Figure 1 demonstrates the specific distribution of the number of observations among biennial CHARLS surveys from 2011 to 2015.

### 2.2. Measures

#### 2.2.1. Internal Migration Experience

The information regarding internal migration experience was from the residence module of the CHARLS life history survey. If the participant had ever left their place of residence (across county boundaries but within national boundaries) for at least 6 months, which is often considered as the threshold of migration or changing place of usual residence in China [36], then the participant was regarded as having “internal migration experience” and the corresponding response was assigned to “Yes”. If the participants had never moved out from their place of residence for 6 months and above, the participant was regarded as having “no internal migration experience” and the corresponding response was assigned to “No”. Overall, 32.1% of the total sample had internal migration experience.

In addition, internal migration frequency and the timing of the first migration experience were also investigated. We measured internal migration frequency by the number of times participants had migrated before the life history survey and divided it into four categories, including no migration experience (67.9%), once (14.6%), twice (10.5%), and three or more times (6.9%). In line with previous studies [21], we grouped ages at initial migration into four categories, including no migration experience (67.9%), 0–17 years old (7.2%), 18–34 years old (18.3%), and 35 years and above (6.6%). 

#### 2.2.2. Depressive Symptoms

The depressive symptoms were measured by the 10-item Center for Epidemiological Studies Depression Scale (CES-D), a widely used self-reported screening tool for depression during the past week (Appendix A Table A1). Each question of the CES-D-10 scale is rated using a four-scale metric, including rarely (<1 day), some days (1–2 days), occasionally (3–4 days), and most of the time (5–7 days). The sum of the 10 items provides a total score of 0 to 30 points, which is consistent with the scoring metric suggested by Radloff [37]. As suggested by Andresen et al. [38], a depression score equal to or above 10 points was used as the cut-off point to identify the presence of depressive symptoms. Accordingly, we generated a dichotomous depression variable (yes = 1, no = 0). A participant was considered to have depressive symptoms if he/she scored no less than 10 points in the CES-D-10. Otherwise, the participant was defined as having no depressive symptoms.

#### 2.2.3. Mediators

Physical health, medical expenditure, pension insurance, and parent–child interaction were regarded as potential mediators underlying the association between internal migration and the presence of depressive symptoms among middle-aged and elderly Chinese.

Physical health was measured by two dichotomous variables (yes = 1, no = 0): chronic disease and physical injury. Chronic disease was scored as 1 if a participant was diagnosed with at least one of the following chronic diseases by the time of interview: hypertension, dyslipidemia, diabetes or high blood sugar, cancer, chronic lung disease, liver disease, heart disease, stroke, kidney disease, stomach disease, psychiatric problems, memory-related diseases, arthritis or rheumatism, and asthma. Physical injury was coded as 1 if a participant ever had a physical injury resulting in permanent handicap, disability, or limitations in daily life. Medical expenditure was measured by the logarithm of per capita household medical expenditure (RMB yuan) in the year before the interview. Accounting for inflation over time, we adjusted the expenditure based on the consumer price indexes (CPI) from 2011 to 2015 in China. Pension insurance was measured as a dichotomous variable (yes = 1, no = 0) and it was coded as 1 if a participant was covered by social pension insurance at the time of the survey. The parent–child interaction was defined by the survey question “How often do you have contact with your children either by phone, text message, mail, or email, when you didn’t live with them?”. Having less than weekly contact with their children was regarded as a low frequency of parent–child interactions and scored as 1, otherwise, the variable was coded as 0. 

#### 2.2.4. Control Variables

Individual, household, and personal life history characteristics were controlled as covariates in our regression analyses. Among them, individual and household characteristics included individual age, gender (male = 1), years of education, marriage status (living with spouse = 1), difficulty in activities of daily living (ADLs) (yes = 1), cognitive function (0–21 points), personal income (RMB yuan, in logarithm form), and family size (number of family members). Difficulty in ADLs was coded as 1 if a participant reported difficulty in performing any of the following six tasks: eating, bathing, dressing, toileting, transferring (e.g., getting into or out of bed, lifting), and continence (control of urination and defecation) [39,40]. Cognitive function was reflected by combining the mental intactness scores (0–11 points) and episodic memory scores (0–10 points) from the survey. Personal income was measured by annual income in the year before the interview, which was also adjusted by the CPI to account for inflation. 

In terms of individual life history characteristics, seven indices were used to measure childhood socioeconomic status (SES) through polychoric principal component analysis (PCA), including the availability of clean cooking fuel (coal/gas/electricity, yes = 1), clean water (tap water, yes = 1), electricity (yes = 1), death of a biological parent (yes = 1), parental occupation (both parents were farmers = 1), parental education (both parents were literate = 1), parental political identity (at least one parent was a communist party member = 1). Childhood health status was self-reported by participants through comparing with their cohorts aged 15 years or younger (much less healthy = 1, somewhat less healthy = 2, about average = 3, somewhat healthier = 4, much healthier = 5). Household registration status was measured using two indicators, including original household registration (*hukou*) status (rural *hukou* = 1) and whether the participants have changed from rural *hukou* to urban *hukou* (yes = 1). In addition, we included survey years in the model to control for the time effect from 2011 to 2015.

### 2.3. Statistical Analysis

Descriptive statistics were calculated for all variables used in this study. Independent sample *t*-tests or chi-square tests were implemented to compare the difference between participants with and without internal migration experience. Multiple logistic regressions were employed to examine the relationship between internal migration experience and depression onset for the overall sample (Model 1) and subsamples stratified by gender, age groups, and types of *hukou*. Specifically, the subsamples included males (Model 2), females (Model 3), middle-aged adults (45–64 years; Model 4), older adults (65 years and older; Model 5), participants who always had rural *hukou* (Model 6), participants who changed from rural *hukou* to an urban one (Model 7), and participants who always had urban *hukou* (Model 8). Following Kim et al. (2021) and Li et al. (2021) [41,42], the multiple logistic regression model was specified as follows:

(1) $$\frac{\pi_{it}}{1-\pi_{it}}=\exp(\beta_{0}+\beta_{1}Mig_{it}+\gamma_{1}X_{1it}+\cdots +\gamma_{k}X_{kit})$$

where $\pi_{it}$ and $(\pi_{it}/1-\pi_{it}$) were the probability and odds of having depressive symptoms for the *i*th participant in period *t*, respectively, $Mig_{it}$ was a dummy variable indicating whether a participant had internal migration experience, and we treated participants who had never experienced migration as the reference group. $X_{1it}\cdots X_{kit}$ represented a set of control variables, including individual, household, and personal life history characteristics, and year dummies. To better interpret the results, we reported odds ratios (OR) to measure how strongly the presence of depressive symptoms was associated with internal migration experience. Using the same model and treating participants with no migration experience as the reference group, we also investigated the associations of internal migration frequency and age at the first migration with depressive symptoms in Model 9 and Model 10, respectively. 

Further, we employed the Karlson–Holm–Breen (KHB) method to explore the possible mechanisms and contributions of potential channels [43]. The KHB method is an unbiased decomposition approach that can be applied to nonlinear probability models to decompose the total effect of a variable into direct and indirect effects and calculate the contributions of each component of potential mediators (indirect effects). In addition, we conducted several robustness checks. On the one hand, to test the robustness of our main findings to the panel data structure, we restricted our sample to the participants who were interviewed and followed in 2011, 2013, and 2015 (balanced panel data) and re-estimated the multiple logistic regression models. On the other hand, we used some proxy indicators regarding psychological status as dependent variables, including CES-D depressions score (Model 12), self-reported health (Model 13), and life satisfaction (Model 14). In these cases, linear regressions (ordinary least square, OLS) and ordered logit models were applied for continuous and ordinal dependent variables, respectively. The statistical significance for all analyses was set at *p* < 0.05, two-sided. All data analyses were conducted using Stata, version 15.1 (StataCorp, College Station, TX, USA). 

## 3. Results

Table 1 describes the summary characteristics of the full sample and two groups defined by migrant status. In the sample, as a whole, the prevalence of depressive symptoms was 34.6%. The mean age and education of the total sample were 59.97 years (SD = 9.56) and 5.30 years (SD = 4.20), respectively, and 48.1% were men. Without considering the confounding effects of control variables, participants who had experienced internal migration (*n* = 14,093) were less likely to suffer from depressive symptoms (33.0% vs. 35.3%, *p* < 0.001) than those without internal migration experience (*n* = 29,761). Meanwhile, participants with internal migration experiences were more likely to be men (*p* = 0.006), older (*p* < 0.001), lived with smaller family sizes (*p* < 0.001), and without a spouse (*p* < 0.001). In comparison with nonmigrants, migrants had higher levels of childhood SES (*p* < 0.001), childhood health status (*p* < 0.001), educational attainment (*p* < 0.001), cognitive function (*p* < 0.001), and personal annual income (*p* < 0.001). In addition, participants who experienced internal migration were less likely to have a rural *hukou* as first *hukou* (85.6% vs. 93.7%, *p* < 0.001) and more likely to change to urban *hukou* (29.5% vs. 18.0%, *p* < 0.001). 

Table 2 presents the association between internal migration experience and depressive symptoms adjusting for individual and household characteristics, as well as early-life conditions. For the full sample regression estimates, internal migration experience was significantly associated with the presence of depression symptoms (OR = 1.07, 95% CI = 1.02–1.12, *p* < 0.01) (Model 1). In terms of gender differences, while internal migration experience had no significant relationship with depression among males (Model 2), it was significantly and positively associated with females’ depression onset (OR = 1.08, 95% CI = 1.01–1.14, *p* < 0.05) (Model 3). In terms of age differences, the results showed that internal migration experience significantly increased the risk of being depressed among the middle-aged participants (OR = 1.12, 95% CI = 1.06–1.19, *p* < 0.001) (Model 4); however, it had no significant effect on the depressive symptoms among older adults (Model 5). For the heterogeneity by the type of *hukou*, the results in Table 3 showed that internal migration experience was significantly and positively linked to the depressive symptoms among rural-to-urban migrants who always had rural *hukou* (OR = 1.13, 95% CI = 1.07–1.19, *p* < 0.001) (Model 6), whereas it was not statistically significant for the depression onset among rural-to-urban migrants who had already obtained an urban *hukou* (Model 7) and urban-to-urban migrants (Model 8). 

Table 4 shows the association of depressive symptoms with migration frequencies (Model 9) and ages at initial internal migration experience (Model 7). The results demonstrated that participants who migrated once (OR = 1.09, 95% CI = 1.02–1.15, *p* < 0.01) and twice (OR = 1.11, 95% CI = 1.03–1.19, *p* < 0.01) had a significantly higher risk to have depressive symptoms than that of participants with no internal migration experience, while participants who experienced migration three or more times had no significant difference in depression compared with nonmigrants (Model 10). In terms of heterogeneity by the age of the first migration, in comparison with participants who had never migrated since birth, respondents who first migrated at 35 years or older had significantly higher probabilities of developing depressive symptoms (OR = 1.14, 95% CI = 1.05–1.25, *p* < 0.01), whereas no significant difference was depicted among those who first migrated below 35 years of age.

As shown in Table 5, the KHB method was employed to explore possible underlying mechanisms of the association between internal migration experience and depressive symptoms. The results suggested that chronic disease (17.98%, *p* < 0.001), physical injury (7.04%, *p* < 0.001), medical expenditure (7.98%, *p* < 0.001), pension insurance (4.91%, *p* < 0.001), and low frequency of interactions with children (4.45%, *p* < 0.01) had statistically significant mediating effects, explaining 42.36% of the total effect (*p* < 0.001). Given that chronic disease had the highest contribution among the mediators proposed in this study, we further investigated which chronic disease was the leading channel underlying the association between internal migration experience and depressive symptoms. Specifically, we used the three most prevalent chronic diseases in our sample, including arthritis or rheumatism (33.4%), stomach or other digestive diseases (22.9%), and hypertension (22.7%), as potential mediators and employed the KHB method to estimate their contributions for the total effects. The results (Appendix A Table A2) showed that arthritis or rheumatism had the strongest mediating effects (10.09%, *p* < 0.001) among the chronic diseases.

Table 6 presents robustness checks for the main findings of this study. First, we generated balanced panel data from 2011 to 2015 and re-estimated the multiple logistic regression for the overall sample (Model 11). The results also showed a significant and positive association between internal migration experience and depressive symptoms (OR = 1.09, 95% CI = 1.03–1.16, *p* < 0.01). Second, we directly used the depression score (CES-D) as the dependent variable and employed a multiple linear regression to examine the association between internal migration experience and depression score (Model 12). The results indicated that internal migration was significantly associated with higher levels of depression among middle-aged and older adults (β = 0.17, 95% CI = 0.05–0.29, *p* < 0.01). Third, given that subjective wellbeing is highly correlated with mental health status [44], we used self-reported health (ranges from “very poor” = 1 to “very good” = 5) and life satisfaction (ranges from “not at all satisfied” = 1 to “completely satisfied” = 5) as dependent variables and applied ordered logit models to investigate the relationship between internal migration experience and subjective wellbeing. As shown in Model 13 and Model 14, internal migration experience was significantly and negatively associated with self-reported health (β = −0.12, 95% CI = −0.16–−0.08, *p* < 0.001) and life satisfaction (β = −0.10, 95% CI = −0.14–−0.06, *p* < 0.001). To sum up, these results suggested that our main findings were robust to the structure of panel data and measures of mental wellbeing.

## 4. Discussion

Using a large representative sample from the CHARLS, this study investigated the association between internal migration experience and depressive symptoms among middle-aged and older adults in China. Toward this end, we first employed multiple logistic regressions to examine whether internal migration experience predicted a higher risk of depression onset for the overall sample. Second, we compared the differences in the mental health consequences of internal migration experience by gender, age group, and type of *hukou*. Third, we examined the heterogeneous effects of different migration frequencies and timing of initial migration on depressive symptoms. Finally, we used the KHB method to explore potential pathways through which internal migration experience affects depressive symptoms, including chronic disease, physical injury, medical expenditure, pension insurance, and parent–child interaction.

This study found that the prevalence of depressive symptoms was 34.6% among middle-aged and elderly Chinese. Internal migration experience was found to be positively associated with participants’ risk of being depressed, which is consistent with our first hypothesis and previous studies regarding the psychological health effects of migration in developing countries [45,46]. A recent study conducted in Mexico also found that domestic migrants reported more anxiety, chronic fatigue, and pain than nonmigrants [47]. Congruent with the second hypothesis, our results showed that, compared with men, older adults, and participants with an urban *hukou*, internal migration experience was more significantly linked to higher risks of depressive symptoms among participants who were women, middle-aged, and rural-to-urban migrants without having an urban *hukou*, respectively. 

Moreover, when considering internal migration frequency, our analyses suggested that participants with low frequency (once and twice) of internal migration experience were more likely to suffer from depressive symptoms, while high migration frequency (three times or above) was not significantly associated with the presence of depressive symptoms. One potential reason is that high migration frequency represents more frequent travels between workplace and hometown, which can reduce the negative effects of family separation due to migrating for work. The CHARLS life history survey has documented the reasons for each time of individual migration. The results showed that “work away (not including the army)” (35.1%), “return to hometown” (24.5%), and, once again, “work away (not including the army)” (46.0%) accounted for the highest proportion among the reasons for the first, second, and third times of internal migration, respectively. This implies that many Chinese internal migrants return to their hometown once in a while to briefly reunite with their family members, and such a family reunion is beneficial to improve migrants’ mental wellbeing [48,49]. In terms of the heterogeneous effects by the age of initial migration, our analysis demonstrated that internal migration experience significantly increased the risk of being depressed among those who firstly migrated at 35 years or older, compared with the younger cohorts. A feasible explanation is that, compared with younger migrants, migrants aged 35 years or older need to take on more family financial responsibilities when they first migrate to cities, such as children’s educational expenditure and economic support for elderly parents [50]. 

In line with the third hypothesis in this study, our mechanism analysis indicated that chronic disease, physical injury, medical expenditure, pension insurance, and parent–child interaction played important mediating roles in the association of internal migration with depressive symptoms among middle-aged and older adults. Meanwhile, chronic disease, especially arthritis or rheumatism, had the largest contribution among the pathways for the total indirect effects, suggesting that the physical health loss due to internal migration could be the leading reason why internal migration experience affects mental health. These results are also consistent with the literature regarding the determinants of the psychological wellbeing of migrants [51,52,53].

Our findings suggest that migration policies should be improved to promote the psychological wellbeing of internal migrants in China. First, actions such as reducing the institutional barriers for nonnative residents are needed to lessen the risk of involuntary split-household arrangements for migrants’ families. Second, preferential social policies and intervention programs are also encouraged for the disadvantaged groups in urban areas, such as female internal migrants and those who have low job skills and high levels of household financial burden. Third, given that overwork status, job security, social insurance (e.g., health insurance and pension insurance), and emotional support are crucial determinants of migrants’ mental health, these dimensions of human wellbeing also deserve policymakers’ attention.

Despite the contribution to the literature about the mental health consequences of internal migration experience in China’s context, this study also has several limitations. First, although the CES-D scale we used has been shown to have a high level of reliability for the measurement of depression, it is a screening tool for depressive symptoms and cannot provide a clinical depression diagnosis. As such, conducting studies with more rigorous clinical diagnostic techniques is encouraged to understand the impacts of migration on psychological wellbeing in China as well as other contexts. Second, due to the data constraints, the potential mediators we proposed and empirically examined in our mechanism analysis may not fully explain the associations between internal migration experience and depressive symptoms. Meanwhile, since the mediating variables used in this study were also extracted from the later-life period, the mechanism analyses in this study should be interpreted as associations rather than causal inferences. Third, our sample only included migrants who were aged 45 and above, and they were not representative for younger cohort migrants, indicating that our findings should be interpreted and generalized with caution. 

## 5. Conclusions

In this study, we found that Chinese middle-aged and older adults with internal migration experience were more likely to develop depressive symptoms than those who never move out from their hometown. This association was more significant among females, middle-aged people, and rural-to-urban migrants who had not obtained an urban *hukou*. We also found that the adverse mental health effect of internal migration could be reduced for those who often reunite with their families and migrate at a younger age with less household financial burden. Association between internal migration experience and the presence of depressive symptoms was shown to be mediated by chronic disease, physical injury, medical expenditure, pension insurance, and parent–child interaction, with the largest contribution of chronic disease. Our findings highlight the necessity and importance of reducing the institutional constraints for internal migration. Evidence-based intervention programs, such as through equalization of health resources and reduction of employment discrimination, as well as social and emotional support, are beneficial to facilitate psychological health among internal migrants.

## Figures and Tables

**Table 1:** Summary characteristics of the participants.

Variables	Full Sample (*n* = 43,854)	With Internal Migration Experience (*n* = 14,093)	Without Internal Migration Experience (*n* = 29,761)	*p*-Value (Chi-square Test/*t* Test)
Depressive symptoms				
No, *n* (%)	28,702 (65.4%)	9438 (67.0%)	19,264 (64.7%)	<0.001
Yes, *n* (%)	15,152 (34.6%)	4655 (33.0%)	10,497 (35.3%)	
Gender				
Female, *n* (%)	22,759 (51.9%)	7180 (50.9%)	15,579 (52.3%)	0.006
Male, *n* (%)	21,095 (48.1%)	6913 (49.1%)	14,182 (47.7%)	
Age (years), Mean (SD)	59.97 (9.56)	60.31 (9.83)	59.81 (9.43)	<0.001
Years of education, Mean (SD)	5.30 (4.20)	6.13 (4.37)	4.91 (4.06)	<0.001
Marriage				
Living without spouse, *n* (%)	7542 (17.2%)	2585 (18.3%)	4957 (16.7%)	<0.001
Living with spouse, *n* (%)	36,312 (82.8%)	11,508 (81.7%)	24,804 (83.3%)	
First *huko*u as rural *hukou*				
No, *n* (%)	3906 (8.9%)	2036 (14.4%)	1870 (6.3%)	<0.001
Yes, *n* (%)	39,948 (91.1%)	12,057 (85.6%)	27,891 (93.7%)	
Having changed to urban *hukou*				
No, *n* (%)	34,344 (78.3%)	9930 (70.5%)	24,414 (82.0%)	<0.001
Yes, *n* (%)	9510 (21.7%)	4163 (29.5%)	5347 (18.0%)	
Childhood SES (PCA score)	−0.36 (0.80)	−0.20 (0.93)	−0.44 (0.72)	<0.001
Childhood health status				
Much less healthy, *n* (%)	2253 (5.1%)	670 (4.8%)	1583 (5.3%)	<0.001
Somewhat less healthy, *n* (%)	3478 (7.9%)	1178 (8.4%)	2300 (7.7%)	
About average, *n* (%)	22,689 (51.7%)	7027 (49.9%)	15,662 (52.6%)	
Somewhat healthier, *n* (%)	8171 (18.6%)	2837 (20.1%)	5334 (17.9%)	
Much healthier, *n* (%)	7263 (16.6%)	2381 (16.9%)	4882 (16.4%)	
Having difficulty in ADLs				
No, *n* (%)	20,795 (47.4%)	6743 (47.8%)	14,052 (47.2%)	0.220
Yes, *n* (%)	23,059 (52.6%)	7350 (52.2%)	15,709 (52.8%)	
Cognitive function, Mean (SD)	10.84 (4.43)	11.54 (4.27)	10.50 (4.47)	<0.001
Personal income (RMB yuan, in logarithm form), Mean (SD)	1.38 (3.36)	1.48 (3.48)	1.33 (3.30)	<0.001
Family size (number of family members), Mean (SD)	3.26 (1.86)	3.17 (1.69)	3.31 (1.93)	<0.001
Year of survey				
2011, *n* (%)	12,978 (29.6%)	4067 (28.9%)	8911 (29.9%)	0.001
2013, *n* (%)	14,177 (32.3%)	4486 (31.8%)	9691 (32.6%)	
2015, *n* (%)	16,699 (38.1%)	5540 (39.3%)	11,159 (37.5%)	

Notes: SD, standard deviation; SES, socioeconomic status; PCA, principal component analysis; ADL, activities of daily living.

**Table 2:** Overall and stratified association between migration experience and depressive symptoms.

	Model 1	Model 2	Model 3	Model 4	Model 5
	Overall	Men	Women	Age: 45–64	Age ≥ 65
	OR (95% CI)	OR (95% CI)	OR (95% CI)	OR (95% CI)	OR (95% CI)
Internal migration experience					
No	Ref.	Ref.	Ref.	Ref.	Ref.
Yes	1.07 (1.02–1.12) **	1.07 (1.00–1.15)	1.08 (1.01–1.14) *	1.12 (1.06–1.19) ***	0.98 (0.90–1.07)
Gender					
Female	Ref.	Ref.	Ref.	Ref.	Ref.
Male	0.75 (0.72–0.78) ***	1.00 (1.00–1.00)	1.00 (1.00–1.00)	0.75 (0.71–0.80) ***	0.72 (0.66–0.78) ***
Age	0.99 (0.98–0.99) ***	0.98 (0.98–0.99) ***	0.99 (0.98–0.99) ***	0.99 (0.99–1.00) **	0.97 (0.96–0.98) ***
Years of education	0.99 (0.98–1.00) *	0.99 (0.98–1.00) *	0.99 (0.98–1.00)	0.99 (0.98–1.00) **	1.01 (1.00–1.02)
Marriage					
Living without spouse	Ref.	Ref.	Ref.	Ref.	Ref.
Living with spouse	0.72 (0.68–0.77) ***	0.64 (0.59–0.71) ***	0.78 (0.72–0.83) ***	0.65 (0.61–0.70) ***	0.79 (0.73–0.87) ***
First *huko*u as rural *hukou*					
No	Ref.	Ref.	Ref.	Ref.	Ref.
Yes	1.25 (1.13–1.37) ***	1.23 (1.06–1.42) **	1.27 (1.12–1.45) ***	1.17 (1.04–1.31) **	1.51 (1.27–1.79) ***
Having changed to urban *hukou*					
No	Ref.	Ref.	Ref.	Ref.	Ref.
Yes	0.83 (0.79–0.88) ***	0.87 (0.80–0.95) **	0.80 (0.75–0.87) ***	0.86 (0.80–0.92) ***	0.78 (0.71–0.87) ***
Childhood SES	0.89 (0.86–0.92) ***	0.87 (0.82–0.92) ***	0.91 (0.87–0.95) ***	0.89 (0.86–0.93) ***	0.93 (0.87–0.99) *
Childhood health status	0.82 (0.80–0.84) ***	0.84 (0.81–0.87) ***	0.80 (0.78–0.83) ***	0.80 (0.78–0.83) ***	0.85 (0.82–0.89) ***
Having difficulty in ADLs					
No	Ref.	Ref.	Ref.	Ref.	Ref.
Yes	3.44 (3.25–3.64) ***	3.50 (3.23–3.78) ***	3.41 (3.15–3.69) ***	3.43 (3.21–3.66) ***	3.40 (3.06–3.78) ***
Cognitive function	0.93 (0.93–0.94) ***	0.93 (0.92–0.94) ***	0.93 (0.92–0.94) ***	0.93 (0.92–0.94) ***	0.93 (0.92–0.94) ***
Personal income	0.96 (0.96–0.97) ***	0.96 (0.95–0.97) ***	0.97 (0.96–0.98) ***	0.96 (0.96–0.97) ***	0.98 (0.96–1.01)
Family size	0.98 (0.97–0.99) **	0.99 (0.97–1.01)	0.98 (0.96–0.99) **	0.98 (0.96–0.99) **	0.99 (0.97–1.01)
Year of survey					
2011	Ref.	Ref.	Ref.	Ref.	Ref.
2013	0.40 (0.38–0.43) ***	0.43 (0.39–0.47) ***	0.38 (0.35–0.42) ***	0.42 (0.39–0.45) ***	0.36 (0.32–0.40) ***
2015	0.42 (0.40–0.45) ***	0.44 (0.40–0.48) ***	0.41 (0.38–0.45) ***	0.44 (0.41–0.47) ***	0.39 (0.35–0.43) ***
Pseudo-R-squared	0.109	0.097	0.090	0.115	0.097
Number of observations	43,854	21,095	22,759	30,577	13,277

Notes: OR, odds ratio; CI, confidence interval; Ref., reference group; SES, socioeconomic status; ADL, activities of daily living. Wald test (*Z* statistic) was performed to check statistical significance; * *p* < 0.05, ** *p* < 0.01, *** *p* < 0.001.

**Table 3:** Association between migration experience and depressive symptoms by type of *hukou*.

	Model 6	Model 7	Model 8
	Always Rural *hukou*	Changed to Urban *hukou*	Always Urban *hukou*
	OR (95% CI)	OR (95% CI)	OR (95% CI)
Internal migration experience			
No	Ref.	Ref.	Ref.
Yes	1.13 (1.07, 1.19) ***	1.01 (0.91, 1.11)	0.95 (0.81, 1.12)
Control variables	Yes	Yes	Yes
Pseudo-R-squared	0.099	0.106	0.083
Number of observations	30,417	9499	3938

Notes: OR, odds ratio; CI, confidence interval; Ref., reference group; Wald test (*Z* statistic) was performed to check statistical significance; *** *p* < 0.001.

**Table 4:** Association between migration experience and depressive symptoms by internal migration frequency and age at the first migration experience.

	Model 9	Model 10
	OR (95% CI)	OR (95% CI)
Frequency of internal migration experience		
No migration experience	Ref.	–
Once	1.09 (1.02–1.15) **	–
Twice	1.11 (1.03–1.19) **	–
Three times or above	1.01 (0.92–1.10)	–
Age at the first migration experience		
No migration experience	–	Ref.
0–17 years old	–	1.08 (0.99–1.18)
18–34 years old	–	1.05 (0.99–1.11)
35 years old or above	–	1.14 (1.05–1.25) **
Control variables	Yes	Yes
Pseudo-R-squared	0.109	0.109
Number of observations	43,854	43,854

Notes: OR, odds ratio; CI, confidence interval; Ref., reference group; Wald test (*Z* statistic) was performed to check statistical significance; ** *p* < 0.01.

**Table 5:** Mechanism analysis using the KHB method.

	Chronic Disease	Physical Injury	Medical Expenditure	Pension Insurance	Low Frequency of Parent–Child Interaction
Estimated value (components of indirect effects)	0.013 (0.008–0.018) ***	0.005 (0.002–0.008) ***	0.006 (0.003–0.009) ***	0.004 (0.002–0.006) ***	0.003 (0.001–0.006) **
Mediating effects (%)	17.98%	7.04%	7.98%	4.91%	4.45%
Total effect	0.073 (0.024–0.123) **				
Direct effect	0.042 (−0.007–0.092)				
Indirect effect	0.031 (0.024–0.038) ***				

*Notes:* 95% confidence intervals in parentheses. Wald test (*Z* statistic) was performed to check statistical significance. ** *p* < 0.01, *** *p* < 0.001.

**Table 6:** Robustness checks of the association between migration experience and depressive symptoms.

	Model 11	Model 12	Model 13	Model 14
	Depressive Symptoms (Balanced Panel)	CES-D Score (OLS)	Self-Reported Health (Ordered Logit)	Life Satisfaction (Ordered Logit)
	OR (95% CI)	Coefficient (95% CI)	Coefficient (95% CI)	Coefficient (95% CI)
Internal migration experience				
No	Ref.	Ref.	Ref.	Ref.
Yes	1.09 (1.03, 1.16) **	0.17 (0.05–0.29) **	−0.12 (−0.16–−0.08) ***	−0.10 (−0.14–−0.06) ***
Control variables	Yes	Yes	Yes	Yes
R-squared/Pseudo-R-squared	0.105	0.176	0.063	0.035
Number of observations	30,372	43,854	43,854	43,854

Notes: Ref., reference group; CI, confidence interval; The *t*-test (*t* statistic) were performed for statistical inference of linear regression in Model 12. Wald test (*Z* statistic) was performed to check the statistical significance of multiple logistic regression and ordered logit regressions in Model 11, Model 13, and Model 14. ** *p* < 0.01, *** *p* < 0.001.

## Data Availability

The CHARLS data can be accessed through its official website (http://charls.pku.edu.cn/index/en.html (accessed on 17 July 2021)).

## Appendix A

**Table A1:** Questions and scoring of the CES-D-10 scale.

Items	Rarely or None of the Time (<1 day)	Some or a Little of the Time (1–2 days)	Occasionally or a Moderate Amount of the Time (3–4 days)	Most or All of the Time (5–7 days)
I was bothered by things that don’t usually bother me				
2. I had trouble keeping my mind on what I was doing				
3. I felt depressed				
4. I felt everything I did was an effort				
5. I felt hopeful about the future				
6. I felt fearful				
7. My sleep was restless				
8. I was happy				
9. I felt lonely				
10. I could not “get going”				
Scoring				
Items 5 and 8	3	2	1	0
All other items	0	1	2	3

**Table A2:** Mechanism analysis using the KHB method: chronic diseases.

	Arthritis or Rheumatism	Stomach or Other Digestive Diseases	Hypertension
Estimated value (components of indirect effects)	0.007 (0.003–0.011) ***	0.002 (−0.001–0.005)	0.000 (−0.001–0.001)
Mediating effects (%)	10.09%	2.76%	0.51%
Total effect	0.073 (0.024–0.123) **		
Direct effect	0.062 (0.015–0.108) **		
Indirect effect	0.009 (0.004–0.015) ***		

Notes: 95% confidence intervals in parentheses. Wald test (*Z* statistic) was performed to check statistical significance. ** *p* < 0.01, *** *p* < 0.001.
